# MicroRNA Signatures for circulating CD133-positive cells in hepatocellular carcinoma with HCV infection

**DOI:** 10.1371/journal.pone.0193709

**Published:** 2018-03-13

**Authors:** Abdel-Rahman N. Zekri, Enas Reda El-Sisi, Amira Salah El-Din Youssef, Mahmoud M. Kamel, Auhood Nassar, Ola Sayed Ahmed, Mohamed El Kassas, Ahmed Barakat Barakat, Alaa Ismail Abd El-Motaleb, Abeer A. Bahnassy

**Affiliations:** 1 Molecular Virology and Immunology Unit, Cancer Biology Department, National Cancer Institute, Cairo University, Cairo, Egypt; 2 Clinical Pathology Department, National Cancer Institute, Cairo University, Cairo, Egypt; 3 Endemic Medicine Department, Faculty of Medicine, Helwan University, Helwan, Egypt; 4 Microbiology Department, Faculty of Science, Ain Shams University, Cairo, Egypt; 5 Surgery Department, Faculty of Medicine, Ain Shams University, Cairo, Egypt; 6 Tissue Culture and Cytogenetics Unit, Pathology Department, National Cancer Institute, Cairo University, Cairo, Egypt; 7 Photobiology and Molecular Biology Department, Laser Institute for Research and Applications (LIRA), Beni-Suef University, Beni Suef, Egypt; University of Kansas School of Medicine, UNITED STATES

## Abstract

**Aim:**

Molecular characterization of the CD133+ stem cells associated with hepatocarinogensis through identifying the expression patterns of specific microRNAs (miRNAs).

**Methods:**

We investigated the expression pattern of 13 miRNAs in purified CD133+ cells separated from the peripheral blood of healthy volunteers, chronic hepatitis C (CHC), liver cirrhosis (LC) and hepatocellular carcinoma (HCC) patients a long with bone marrow samples from the healthy volunteers and the LC patients using custom miScript miRNA PCR array.

**Results:**

The differential expression of the 13 studied miRNAs in CD133+ cells separated from the HCC patients' peripheral blood compared to the controls revealed that *miR-602*, *miR-181b*, *miR-101*, *miR-122*, *miR-192*, *miR-125a-5p*, and *miR-221* were significantly up regulated (fold change = 1.8, 1.7, 2, 5.4, 1.6, 2.9 & 1.5 *P* value = 0.039, 0.0019, 0.0013, 0.0370, 00024, 0.000044 &0.000007 respectively). As for the HCC group compared to the CHC group; *miR-602*, miR-122, *miR-181b*, *miR-125a-5p*, and *miR-192* were significantly up regulated (fold change = 13, 3.1, 2.8, 1.6 & 1.56, *P* value = 0.01, 0.001, 0.000004, 0.002 & 0.007 respectively). Upon comparing the HCC group to the LC group; *miR-199a-3p*, *miR-192*, *miR-122*, *miR-181b*, *miR-224*, *miR-125a-5p*, and *miR-885-5p* were significantly up regulated (fold change = 5, 6.7, 2.3, 3, 2.5, 4.2 & 39.5 *P* value = 0.001025, 0.000024, 0.000472, 0.000278, 0.000004, 0.000075 & 0.0000001 respectively) whereas *miR-22* was significantly down regulated (fold change = 0.57 *P* value = 0.00002). Only, *miR-192*, *miR-122*, *miR-181b* and *miR-125a-5p* were significant common miRNAs in CD133+ cells of the HCC group compared to the other non-malignant groups.

**Conclusion:**

We identified a miRNA panel comprised of four miRNAs (*miR-192*, *miR-122*, *miR-181b* and *miR-125a-5p*) that may serve as a molecular tool for characterization of the CD133+ cells associated with different stages of hepatocarinogensis. This panel may aid in developing a new target therapy specific for those CD133+ cells.

## Introduction

Hepatocellular carcinoma (HCC) is the most common primary liver cancer and the second leading cause of the cancer-related death in the world. Also, It ranks as the fifth and seventh common cancer in men and women respectively **[1].** Egypt is one of the countries who has the highest prevalence of hepatitis C virus (HCV) worldwide estimated by (21.9%) among adults in 1995–1996 **[2].**

CD133+ is a transmembrane glycoprotein that known as an important cell surface marker of the putative normal liver stem cells “oval cells” as well as cancer stem cells (CSCs) **[3–6].** In line with this, the increased expression of CD133+ was found to be correlated with advanced stages of HCC **[7,8].** A special attention is given to the naive circulating CD133+ cells and its clinical significance in HCC prognosis. We have previously showed that circulating CD133+ cells were significantly higher in the blood samples of chronic hepatitis and HCC patients compared to the healthy controls; revealing the prognostic role of CD133+ cells in those patients **[9]**.

MicroRNAs (miRNAs) are small, non-coding RNAs that can regulate carcinogenesis and cancer development through their complementary binding to mRNAs of oncogenes or tumor suppressors genes **[10,11]**. miRNAs are involved in the regulation of proliferation and self-renewal of the CSCs and they also promote the differentiation to determine the fate of stem cell **[12]**. Both normal stem cells (NSCs) and CSCs are regulated by miRNAs which accounts for the importance of the identifying the most common and unique miRNAs expression patterns between CSCs and NSCs.

The purpose of this study is to assess the expression patterns of 13 miRNAs in the naive circulating CD133+ stem cells associated with different stages of hepatocarcinogensis on top of HCV infection. Those miRNAs have been previously reported in literature to play a role in hepatocarcinogensis machinery. Also, this study is considered as a complementary study to our previously published paper which illustrated the expression pattern of those miRNAs in the serum samples of CHC, LC and HCC patients on top of HCV infection as well as healthy controls **[13, 14].**

## Patients and methods

### Study design

This retrospective case control study was conducted on thirty subjects who attended the National Cancer Institute, Cairo University. The recruited patients were categorized into five groups; 1) six donors for bone marrow (BM) transplantation who were enrolled as control group; 2) nine chronic hepatitis C (CHC) patients; 3) six patients with liver cirrhosis (LC) and 4) nine HCC patients 5) additional 16 blood samples act as confirmatory set (8 HCC and 8 Control samples). All control subjects had normal serum alanine aminotransferase (ALT) level and all were seronegative for HBV and HCV antibodies. The CHC and LC patients were diagnosed by clinical examination, abdominal ultrasound, laboratory investigations and liver biopsy. The diagnosis of HCC patients was based on computed tomography (CT) and elevated AFP level and confirmed by histopathology. The criteria for inclusion were as follows: a) adult patients of both sexes who were positive for HCV antibodies (EIAgen HCV Ab (v.4) kit code: 071064, adaltis. Milano Italy) as well as HCV RT-PCR (*artus* HCV RT-PCR Kits CE, cat no. 4518265, QIAGEN GmbH, QIAGEN Strasse 1, D-40724 Hilden); b) newly diagnosed HCC cases without prior chemotherapy. The criteria for exclusion were as follows: a) patients with positive hepatitis B surface antigen (HBsAg) and was confirmed by PCR; b) patients who received previous treatment for HCC. The study was approved by the Institutional Review Boards (IRB) of the National Cancer Institute, Cairo University, which was in accordance with the guidelines of 2004 Declaration of Helsinki. A written informed consent was obtained from all participants prior to enrollment in the study (Organization No.IORG0003381. IRB NO.IRB00004025).

### Collection of clinical specimen

Ten milliliters of venous peripheral blood were collected from all enrolled subjects in Cell- Save blood collection tubes (Immunicon Inc., Huntingdon Valley, PA, United States) containing a cellular preservative and EDTA. Similarly, 10 ml of heparinized BM aspirate were collected from LC patients and normal volunteers during their regular treatment regimen and follow up. Briefly, patients' skin was cleaned with 70% ethanol at the iliac crest with a boring movement. Then, needles were passed perpendicularly into the cavity of the ileum at a point just posterior to anterior superior iliac spine or 2 cm posterior and 2 cm inferior to the anterior superior iliac spine to aspirate the BM (Salah and Klima New Delhi 110055, Delhi, India). The mononuclear cells were isolated from freshly collected peripheral blood and BM samples using RBC's lysis buffer. Cells were washed twice using phosphate buffer saline and counted using a hemocytometer.

### CD133+ cells' sorting

CD133^+^ cells were isolated using MACS kit (Miltenyi Biotec, Germany) according to manufacturer's instructions. The mononuclear cells were incubated for 30 min. at 4°C after labeling with 100μl CD133/1 magnetic microbeads and 100 μl FcR. Then, the cells were sorted twice using the autoMACS™ separator (Miltenyi Biotec, GermanyF) to increase their purity. The cells were washed twice with phosphate buffered saline (pH 7.2); supplemented with 0.5% bovine serum albumin and 2mM EDTA, and centrifuged at 300 xg for 10 min at 4°C. For confirmatory set, CD133+ cells were sorted using the BD FACSCalibur™ (BD Biosciences, USA) platform that allows performing both cell analysis and cell sorting in a single bench top system.

### Flow cytometry analysis

The sorted cells were incubated with phycoerythrin (PE)-conjugated anti-CD133/2 for 30 min at 4°C. (Miltenyi Biotec, Bergisch Gladbach, Germany). Then, they were treated with FcR blocking reagent (Miltenyi Biotec). The labeled cells were analyzed with Flowcytometer Epics® Elite Coulter system using the CELL Quest software (Becton–Dickinson Immunocytometry Systems, San Jose, CA, USA). A nonspecific isotype control was used in each sample. All antibodies were of IgG1k isotype. Results were expressed as a specific percentage of positive markers, calculated by subtracting the nonspecific fluorescence of the isotype control form the specific fluorescence of the monoclonal antibody.

### RNA extraction and cDNA synthesis

Total RNA including miRNA was extracted using RNeasy mini kit (Qiagen cat. no. 217004) and QIAzol lysis reagent according to manufacturer’s instructions. The quality of the RNA was estimated using 2000\2000c nanodrop (Thermo scientific, USA). RT2 miRNA First Strand Kit (Qiagen) was used to prepare cDNA from 500 ng RNA. Reverse transcription was carried out in a final reaction volume of 20 μl that were incubated for 60 min at 37°C followed by 5 min at 95°C.

### Quantitative real-time PCR

The relative expression levels of 13 mature miRNAs [hsa-miR-122, hsa-miR-192, hsa-miR-885-5p, hsa-miR-375, hsa-miR-224, hsa-221, hsa-miR-22, hsa-miR-101, hsa-miR-602, hsa-miR-125a-5p, hsa-miR-181b, and hsa-miR-199a-3p] in CD133 cells were performed using miScript miRNA PCR custom array (SABiosciences, custom array catalog number caih0038). For real time PCR, the reaction mixture contained 13 μl SYBR PCR master mix and 2 μl diluted cDNA (1:10) in a final volume of 25 μl for each miRNA. The amplification step was carried out for 40 cycles according to the following conditions: 95°C for 10 minutes, 1 cycle; 95°C for 15 seconds, 60°C for 40 seconds, 72°C for 30 seconds. The 13 miRNAs were compared and analyzed across multiple plates using the ΔΔCt method and normalized to *snord48* (housekeeping gene).

### miRNAs selection and target prediction

The selection of the miRNAs was based on a previous review of literature along with using miR2Disease database; a comprehensive resource of miRNA deregulation in various human diseases (http://www.mir2disease.org). While, miRTaBase database; the experimentally validated miRNA-target interaction database *(mirtarbase*.*mbc*.*nctu*.*edu*.*tw*) was used to predict downstream targets of miRNAs.

### Confirmatory reverse transcription and Quantitative Real-Time Polymerase Chain Reaction (qRT-PCR)

CD133 cells were sorted from blood samples obtained from eight HCC patients and eight control subjects using flowcytometry with cell sorter. Total RNA was reverse transcribed for sorted CD133 cells using miScript II RT kit (Qiagen). Reactions were incubated at 37°C for 1 hr followed by inactivation of the reaction by incubation at 95°C for 10 min. For miRNA expression Confirmatory Real-time PCR was carried out for the four miRNAs (miRNA-122, miRNA181b, miRNA-125a-5p, miRNA-192) using primers specific for these miRNAs, **Table 1**, 1 μL of diluted RT product was used (equivalent to 3 ng) as template in a 10 μL PCR reaction containing 1X SYBR Green master mix (Qiagen), 200nMmiRNA specific forward primer, and 200 nM universal primer. The conditions for qRT-PCR were as follows: 95°C for 10min, followed by 40 cycles of 95°C for 15s and 50°C for 30 s and 70°C for 30 s. All the RT-qPCR reactions were performed on 7500 fast real-time PCR system (Applied Biosystems). All samples were analysed in duplicate.

**Table 1:** The primer sequence of the four miRNAs used for the confirmatory set.

miRNA	Sequence
hsa-miR-122	UGGAGUGUGACAAUGGUGUUUG
hsa-miR-192	CUGACCUAUGAAUUGACAGCC
hsa-miR-125a-5p	UCCCUGAGACCCUUUAACCUGUGA
hsa-miR-181b	AACAUUCAUUGCUGUCGGUGGGU

### Statistical analysis

Data analysis was performed using the supplied software "http://www.sabiosciences.com/pcrarraydataanalysis.php"; based on Student’s t-test of the replicate (2^ (-ΔCt) values for each miRNA in the tested groups and the control group. P-values less than 0.05 are considered statistically significant. Fold-change values >1 imply an up-regulation while fold-change values <1 imply a down-regulation. The SPSS software package (version 15) was used to analyze the clinical data. Continuous variables were expressed as median and range while categorical variables were expressed as percentages. Comparisons between groups were analyzed by one way ANOVA for the continuous variables and by the χ2 or Fisher’s exact test for the categorical variables whenever possible.

## Results

### The demographic features and the clinical data of the studied groups

The demographic and the clinical features of the different studied groups were shown in **Table 2.** The age differed significantly between the studied groups **(*P* value < 0.05)** with a trend for increasing ages with disease progression. However, there was no significant difference between the LC and the HCC groups regarding the age. Also, no significant difference was observed regarding the gender between the studied groups with male predominance in the groups with HCV-related liver disease except for those in the CHC group. The level of AST was significantly higher in the LC and CHC groups compared to the other groups (***P* value < 0.05**). However, levels of total bilirubin and AST were significantly higher in the LC group compared to the other groups **(*P* value < 0.05, 0.01 respectively)**. Albumin was significantly lower in the LC group compared to the other groups **(*P* value < 0.05)** whereas; AFP was significantly higher in the HCC group compared to the other groups **(*P* value < 0.01)**.

**Table 2:** The clinical data of the studied groups.

	Control (n = 6)	Non cirrhotic (n = 9)	Cirrhotic (n = 6)	HCC (n = 6)	*P* value
**Age**					**<0.05**
Median	30.5 a	42 b	56 c	64 c	
Range	(26–34)	(28–58)	(43–63)	(49–88)	
**Gender**					0.07
Male	5 (83%) a	4 (44%) a	4 (67%) a	4 (67%) a	
Female	1(17%) a	5 (56%) a	2 (33%) a	2 (33%) a	
**ALT**					**<0.05**
Median	12 a	47 b	45 b	25.5 ab	
Range	(7–22)	(31–83)	(34–145)	(12.9–74)	
**AST**					**<0.05**
Median	15 a	39 b	70 c	38.5 b	
Range	(12–36)	(24–64)	(59–134)	(35–137)	
**T.Bilirubin**					**<0.01**
Median	0.6 a	0.9 a	2.78 c	1.35 a	
Range	(0.5–0.9)	(0.3–1.92)	(1.4–6.52)	(0.37–2)	
**Albumin**					**<0.05**
Median	3.5 a	4.6 b	2.6 c	3.5 a	
Range	(3.0–4.6)	(4.0–4.9)	(2.0–3.0)	(2.5–4.2)	
**HCV Ab**					**<0.01**
Present	0 (0%) a	9 (100%) b	6 (100%) b	6 (100%) b	
Absent	6 (100%) a	0 (0%) b	0 (0%) b	0 (0%) b	
**AFP**					**<0.01**
Median	1.2 a	5.7 b	5.8 b	297 c	
Range	(0.2–3.5)	(0.8–12.7)	(3.6–6.0)	(18.2–1274.7)	

Groups bearing different initials are significantly different."ALT" = alanine aminotransferase, "AST" = aspartate aminotransferase, "AFP" = alpha feto protein, "T-Biliribin" = total bilirubin

### CD133+ subpopulation purity in the different studied groups

**Fig 1:**
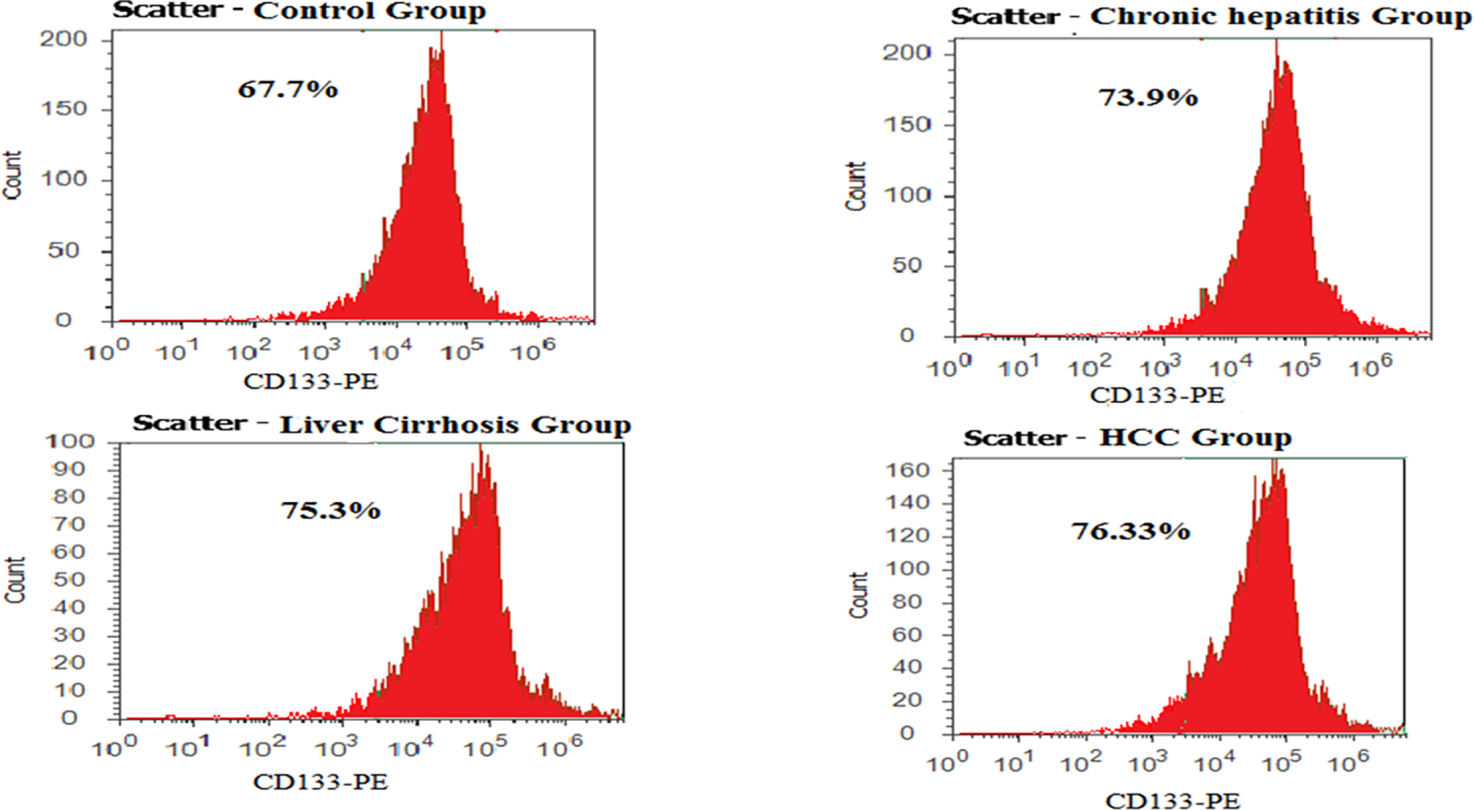
The percentage of CD133+ cells in the different studied groups. After sorting by autoMACS™ (Miltenyi Biotec), the percentage of CD133+ fractions ranged from 67–76%.

Prior to performing the custom miScript miRNA PCR array, the purity of sorted CD133+ cells was tested in all studied groups by flowcytometry. The purity of CD133+ subpopulation ranged from 67 to 76% in the different studied groups as shown in **Fig 1**.

### The differential expression of miRNAs in CD133+ cells purified from the peripheral blood (PB) of the different studied groups

**Fig 2:**
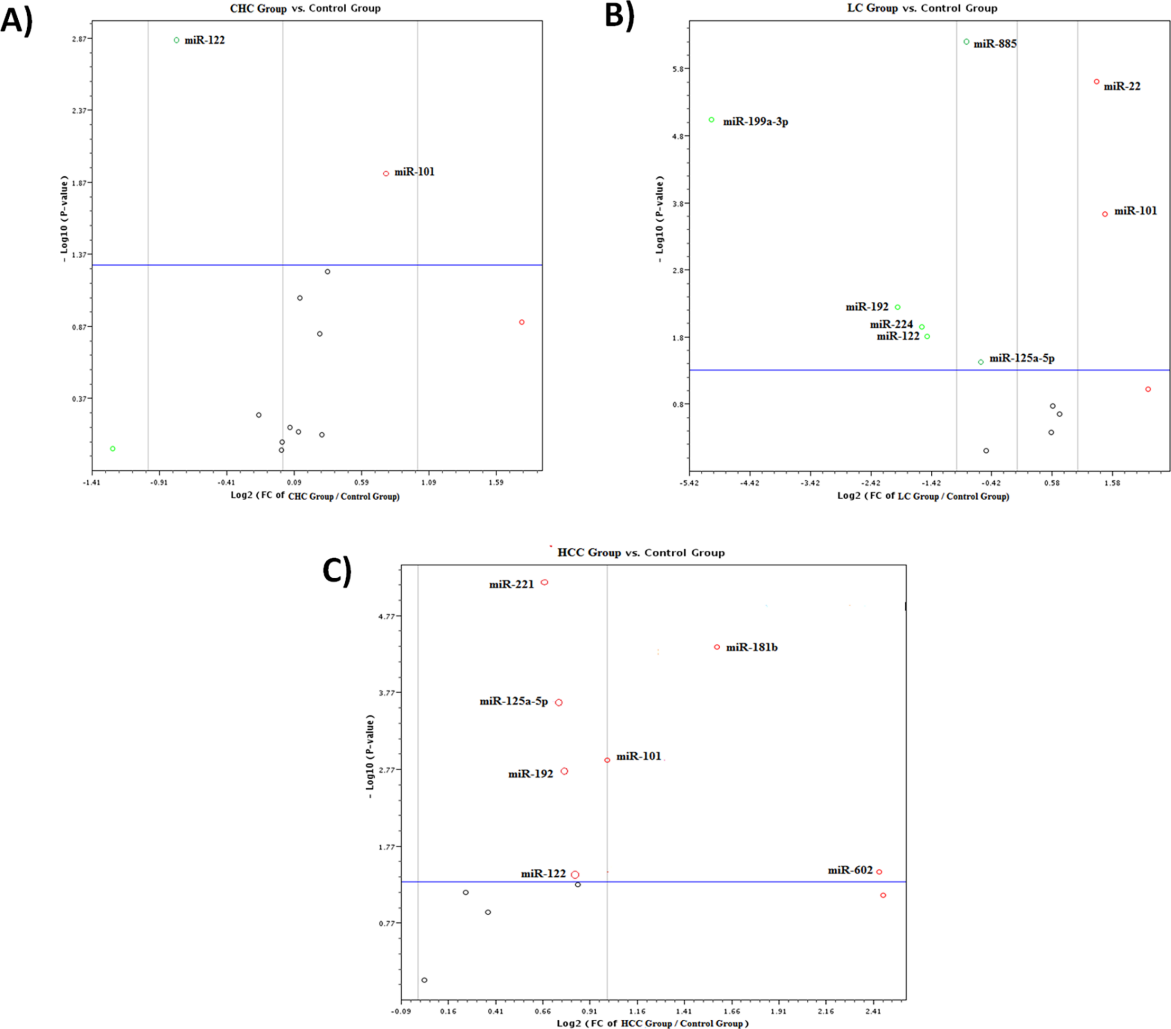
A) Volcano plot representing the differential expression of the 13 miRNAs in CD133+ cells of the CHC group (PB) versus the control group (PB). B) Volcano plot representing the differential expression of the 13 studied miRNAs in CD133+ cells of the LC group (PB) versus the control group (PB). C) Volcano plot representing the differential expression of the 13 miRNAs in the CD133+ cells of the LC group (PB) versus the control group (PB).

Among the 13 studied miRNAs, only *miR-101* was significantly up regulated (fold change = 1.69 *P* value = 0.016) while *miR-122* was significantly down regulated (fold change = -1.74 *P* value = 0.0114) upon comparing CD133+ cells of the CHC group to those of the control group as shown in **S1 Table** and **Fig 2A**. Moreover, *miR-22* and *miR-101* were significantly up regulated (fold change = 2.4 & 2.7 *P* value = 0.000002 &0.0002 respectively) whereas *miR-122*, *miR-192*, *miR-885-5p*, *miR-224*, *miR-125a-5p* and *miR-199a-3p* were significantly down regulated (fold change = 0.36, 0.25, 0.55, 0.33.0.66 & 0.03 *P* value = 0.000009, 0.005, 0.011, 0.015, 0.000001 & 0.03 respectively) on comparing CD133+ cells of the LC group to the control group as shown in **S2 Table** and **Fig 2B.** Furthermore, *miR-122*, *miR-192*, *miR-101*, *miR-602*, *miR-125a-5p*, *miR-181b* and *miR-221* were significantly up regulated (fold change = 1.8, 1.7, 2, 5.4, 1.6, 2.9 & 1.5 *P* value = 0.039, 0.0019, 0.0013, 0.0370, 00024, 0.000044 &0.000007 respectively) upon comparing CD133+ cells of the HCC group to the control group as shown in **S3 Table** and **Fig 2C.**

**Fig 3:**
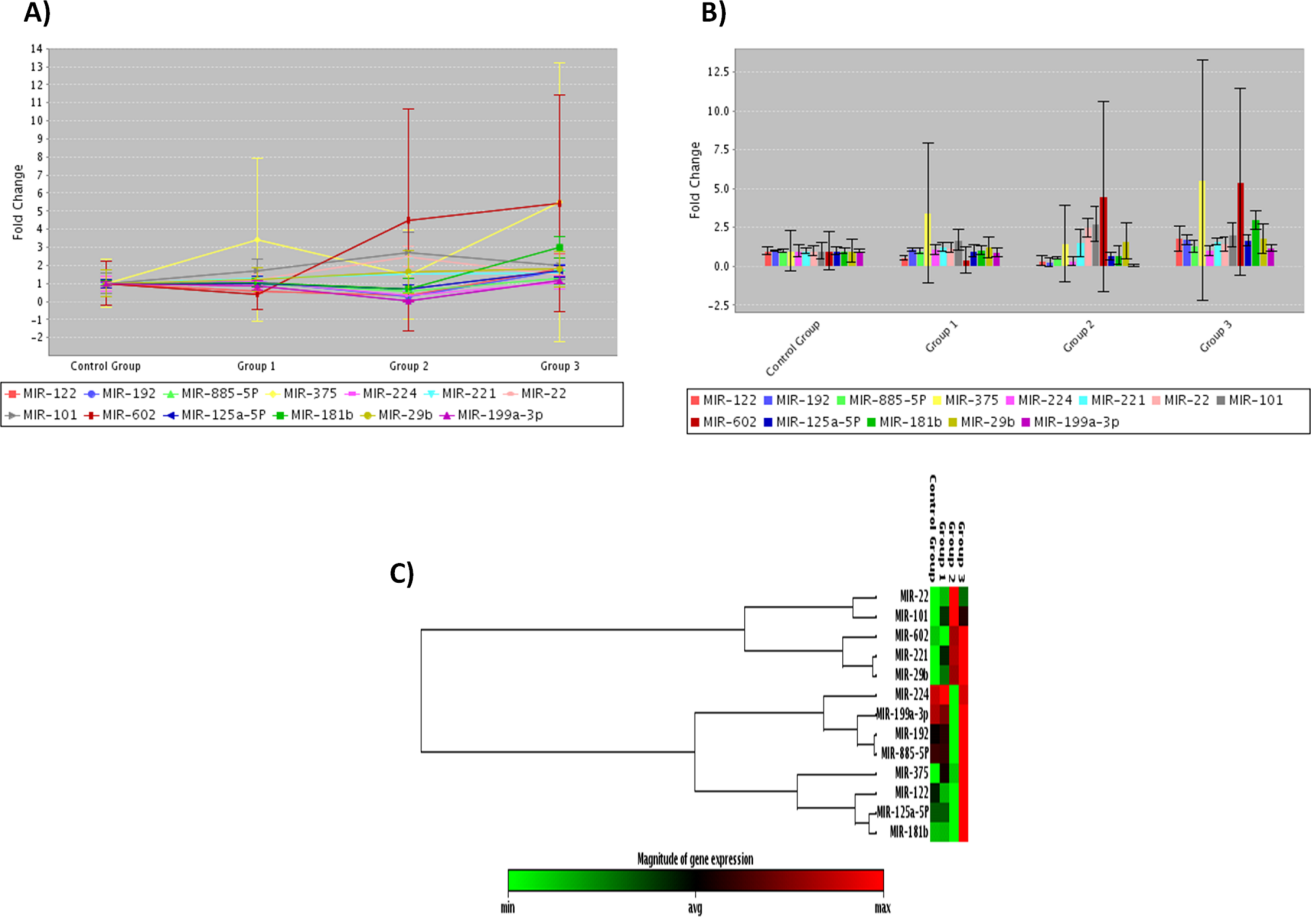
A) The multi group plot provided both a bar chart and line graph representation (with optional error bars) for differential expression of the13 miRNAs in CD133+ cells of the three groups (PB) compared to control group (PB) where group 1 represented CHC group, group 2 represented LC group and group 3 represented HCC group. B) The heat map with dendrograms representing co-regulated miRNAs across all groups.

The heat map with dendograms for the differential expression of 13 miRNAs in CD133+ cells of the three studied groups compared to the control group indicates co-regulated miRNAs across all studied groups along with multi group plot provided both a bar chart and line graph representation (with optional error bars) are shown in **Fig 3.**

**Fig 4:**
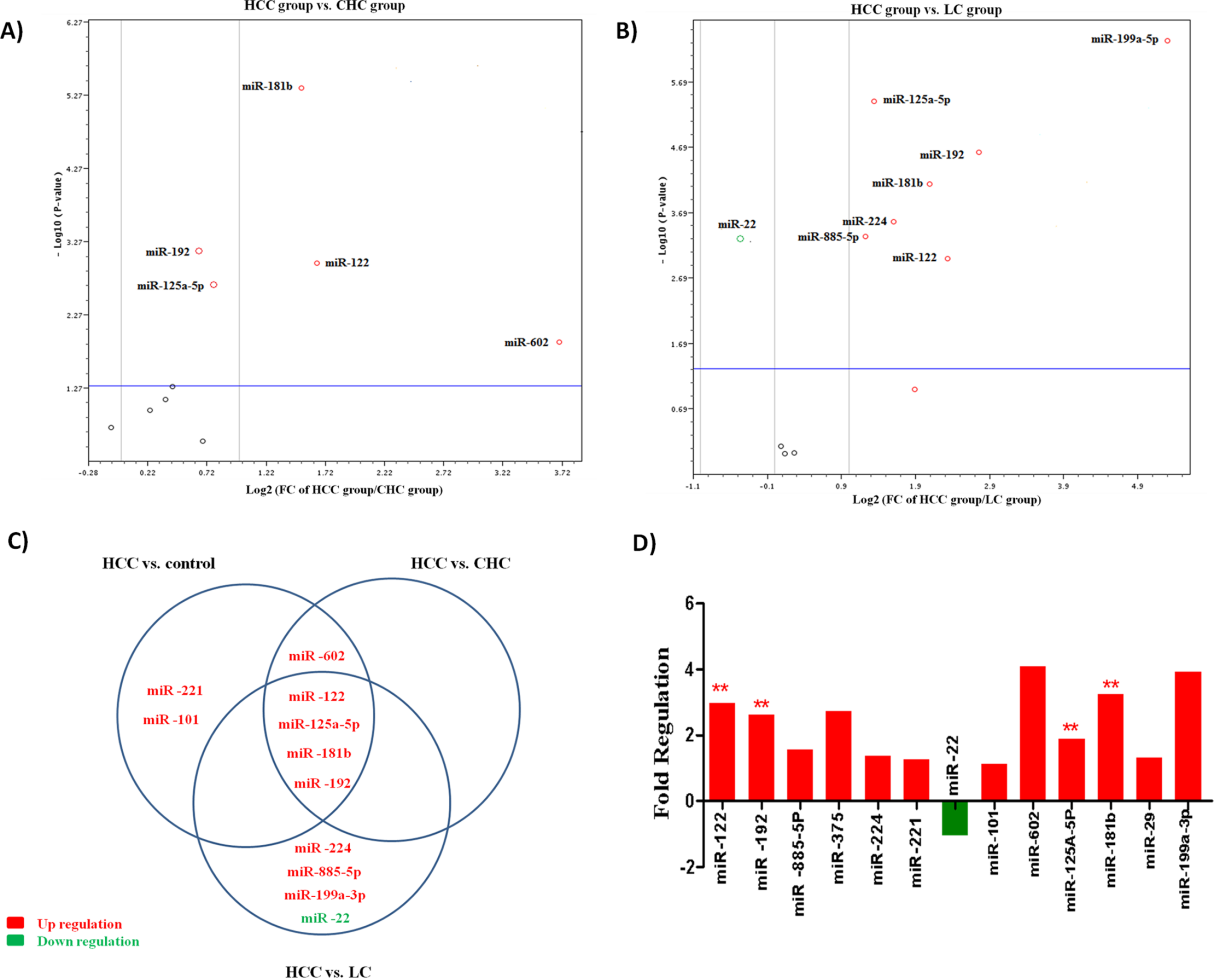
A) Volcano plot representing the differential expression of the 13 miRNAs in CD133+ cells of the HCC group (PB) versus the CHC group (PB). B) Volcano plot representing the differential expression of the 13 miRNAs in CD133+ cells of the HCC group (PB) versus the LC group (PB). C) Venn graph represented the differential expression of the 13 miRNAs in the CD133+ of HCC group (PB) in comparison with the control, CHC, and LC groups. D) Histogram showing the differential expression of the 13 miRNAs in CD133+ cells of the HCC group (PB) versus other non-malignant groups (PB)."*" miRNA is significant at 0.05 level while "**" miRNA is significant at 0.01 level.

Among the 13 studied miRNAs in CD133+ cells of the HCC group compared to the CHC group; *miR-602*, *miR-122*, *miR-181b*, *miR-125a-5p* and *miR-192* were significantly up regulated (fold change = 13, 3.1, 2.8, 1.6 & 1.56 *P* value = 0.01, 0.001, 0.000004, 0.002 & 0.007; respectively) as shown in **S4 Table** and **Fig 4A.** On comparing CD133+ cells of the HCC group to the LC group; *miR-122*, *miR-192*, *miR-885-5p*, *miR-224*, *miR-125a-5p*, *miR-181b* and *miR-199a-3p* were significantly up regulated (fold change = 5, 6.7, 2.3, 3, 2.5, 4.2 & 39.5 *P* value = 0.001025, 0.000024, 0.000472, 0.000278, 0.000004, 0.000075 & 0.0000001 respectively) whereas *miR-22* was significantly down regulated (fold change = 0.57 *P* value = 0.00002) as shown in **S5 Table** and **Fig 4B**. The differential expression of 13 miRNAs in CD133+ cells of the HCC group compared to the other studied groups was illustrated in **Fig 4C** and **Fig 4D**.

It has been shown that *miR-122*, *miR-192*, *miR-125a-5p* and *miR-181b* were significantly common up regulated miRNAs in CD133+ cells of the HCC group (fold change = 5, 6.7, 2.3 & 39.5, *P* value = 0.001025, 0.000024 & 0.0000001; respectively) up on comparing CD133+ cells of the malignant group (HCC group) to the other non-malignant groups (CHC, LC and control groups) as shown in **S6 Table.**

**Fig 5:**
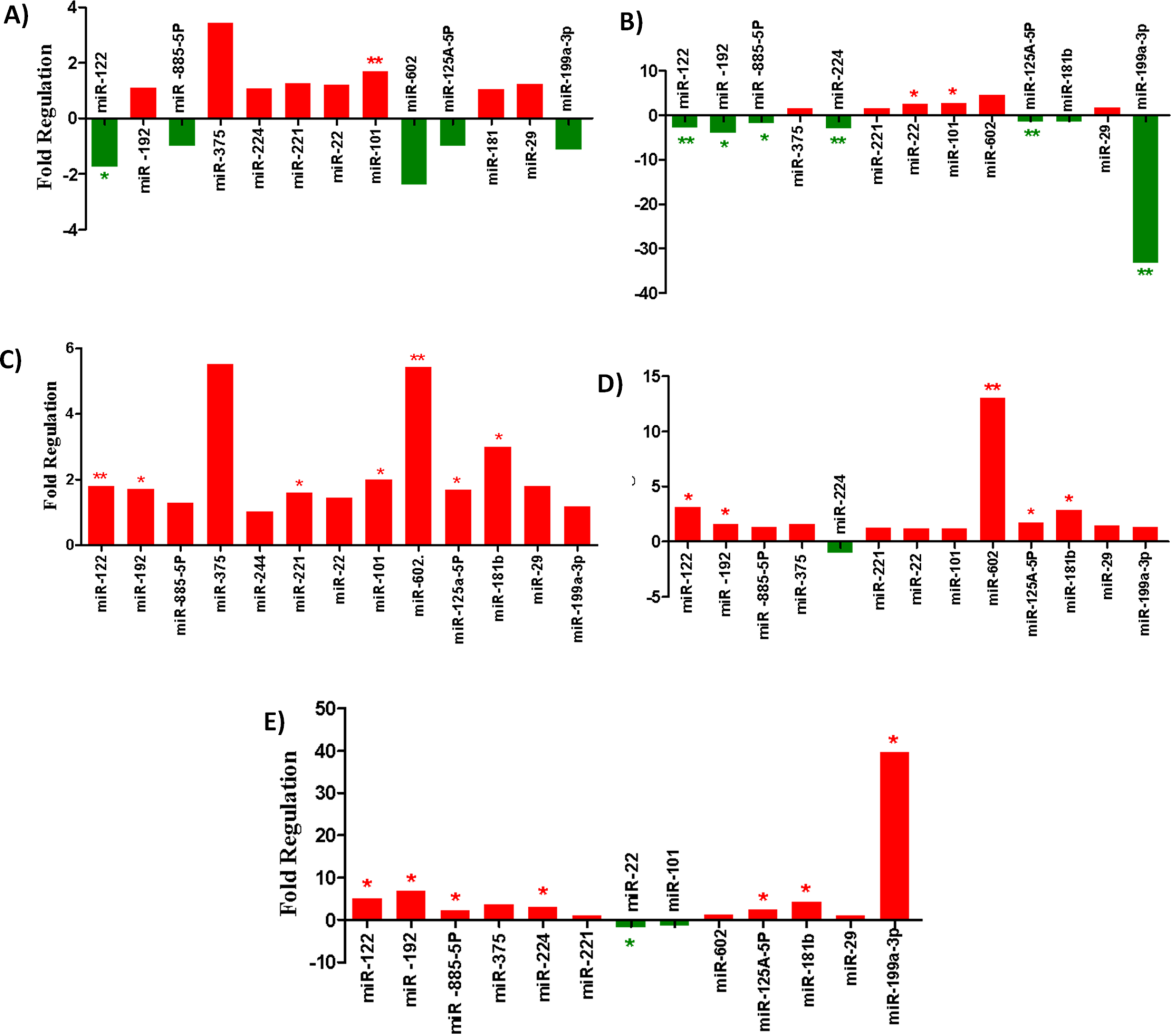
A) Histogram showing the differential expression of the 13 miRNAs in CD133+ cells the CHC group (PB) versus the control group (PB). B) Histogram showing the differential expression of the 13 miRNAs in CD133+cells the LC group (PB) versus the control group (PB). C) Histogram showing the differential expression of the 13 miRNAs in CD133+ cells of the HCC group (PB) versus the control group (PB). D) Histogram showing the differential expression of the 13 miRNAs in CD133+ of the HCC group (PB) versus the CHC group (PB). E) Histogram showing the differential expression of the 13 miRNAs in CD133+ of the HCC group (PB) versus the LC group (PB). "*" miRNA is significant at 0.05 level while "**" miRNA is significant at 0.01 level.

The histograms representing the differential expression of miRNAs in CD133+ cells purified from peripheral blood of all studied groups were shown in **Fig 5**.

**Fig 6:**
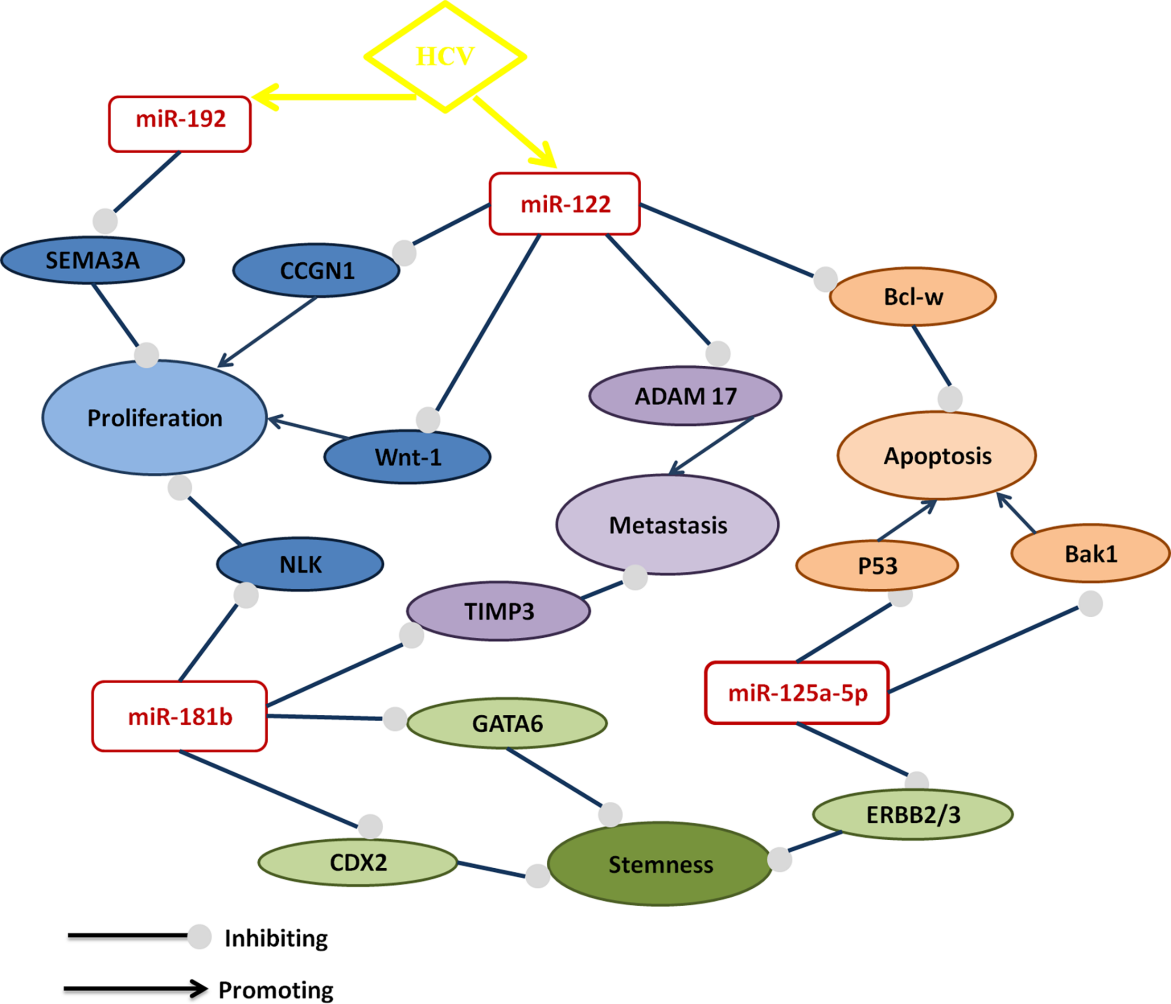
Suggested cartoon representing the relationship of significant overlapping miRNAs in CD133+ stem cells associated with hepatocarinogensis on top of HCV infection. The green color of miRNAs indicates negative fold regulation while and red color indicates positive fold regulation.

The possible mechanistic role of significant overlapping miRNAs in CD133+ cells of HCC with their downstream targets as well as the relationship between miRNAs and HCV were illustrated in **Table 3** and **Fig 6**.

**Table 3:** Functional annotation of miRNAs and their target genes in CD133+ cells of HCC.

miRNA	Target	Gene annotation
***miR-122***	*Bcl-w*	Inhibits apoptotic pathway
	*ADAM17*	Process many receptors as EGFR, TNFR- I and TNFR-II Notch receptors. It plays a prominent role in notch pathway activation
	*Wnt-1*	Reacts with frizzled family receptor results in activation of wnt/β-catenin.
	*CCNG1*	p53 and p73 activate the CCNG1 during the transcription steps that in turn suppress p53 family proteins leading to cell survival activation.
***miR-192***	*SEMA3A*	Inhibits the Rac-PAK pathway responsible for regulation of actin dynamics; resulting in cytoskeleton remodeling.
***miR-181b***	*TIMP3*	Exerts its anti-angiogenic effect via a direct interaction with vascular endothelial growth factor (VEGF) receptor-2 resulting in inhibition of proliferation, migration and tube formation of endothelial cells (ECs).
	*GATA6*	The transcription factor GATA6 directly regulates expression of leucine-rich repeat containing G protein-coupled receptor 5 (LGR5) involved in the differentiation pathway.
	*CDX2*	CDX2 is (caudal type homeobox 2) is an important transcription factor in the differentiation pathway
	*NLK*	Inhibits of wnt/β-catenin signaling
***miR-125a-5p***	*BAK-1*	Involved in S6K1-BAD-BAK apoptotic pathway
	*P53*	Involved in P53 apoptotic pathway
	*EBBR2/3*	Involved in cytoskeleton remodeling

### The differential expression of miRNAs in CD133+ cells purified from the bone marrow (BM) of the different studied groups

**Fig 7:**
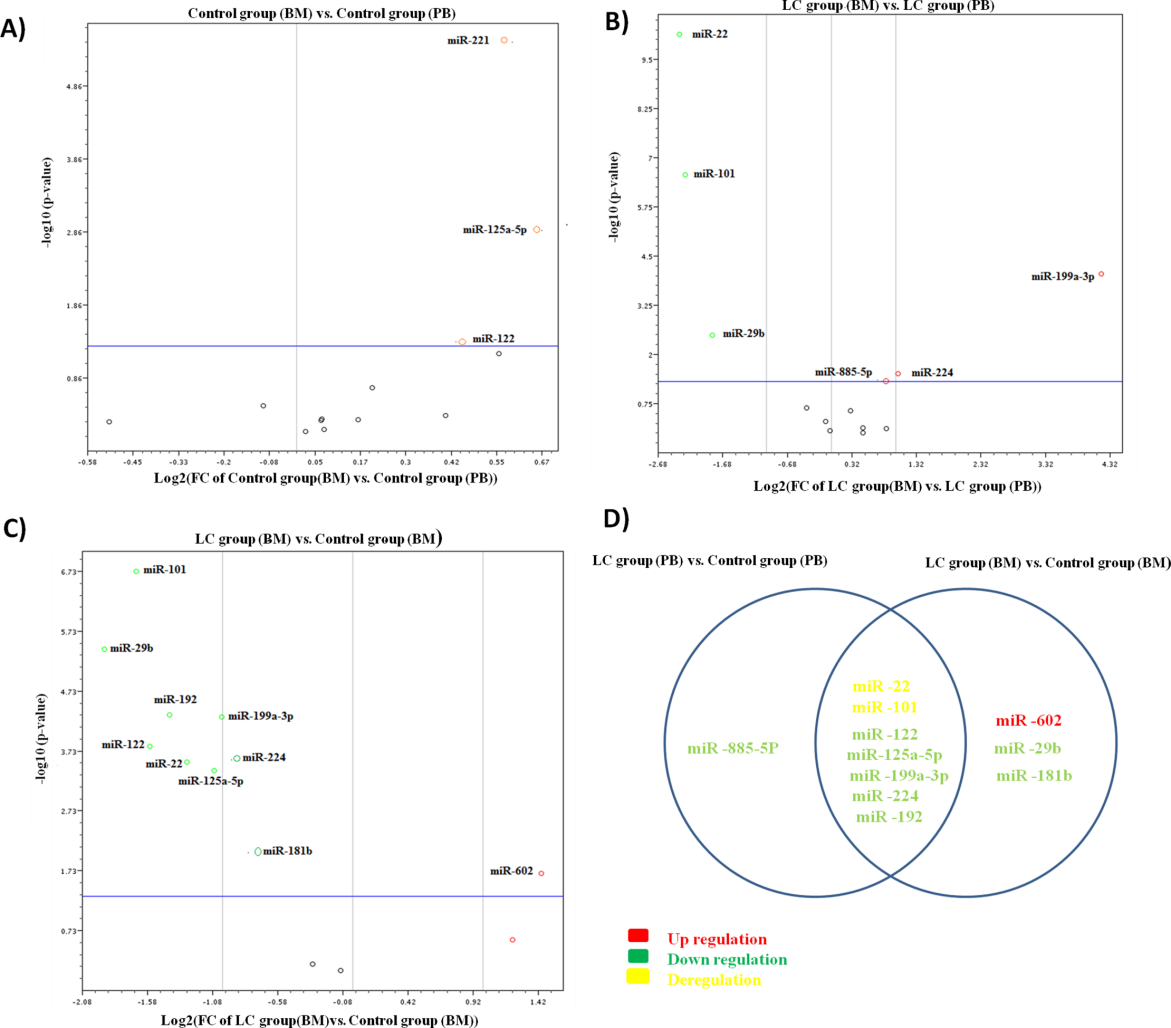
A) Volcano plot representing the differential expression of the 13 miRNAs in CD133+ cells of the control group (BM) versus the control group (PB). B) Volcano plot representing the differential expression of the 13 miRNAs in CD133+cells of the LC group (BM) versus the LC group (PB). C) Volcano plot representing the differential expression of the 13 miRNAs in CD133+ cells of the LC group (BM) versus the control group (BM). D) Venn graph showing the differential expression of 13 miRNAs of the CD133+ cells of the LC group (PB&BM) compared to the CD133+ of control group (PB & BM) respectively.

The differential expression of the studied miRNAs in CD133+ cells separated from BM to those separated from PB of the control group revealed that only 3 out of 13 miRNAs were significantly up regulated; *miR-122*, *miR-221* and *miR-125a-5p* (fold change = 1.4, 1.5 & 1.6 *P* value = 0.044, 0.000004 & 0.001312 respectively) as shown in **S7 Table** and **Fig 7A.** Furthermore, *miR-885-5p*, *miR-224* and *miR-199a-3p* were significantly up regulated (fold change = 1.74, 2 & 18.2 *P* value = 0.048477, 0.031087&0.000091 respectively) whereas *miR-22*, *miR-101* and *miR-29b* were significantly down regulated (fold change = 0.19, 0.2 & 0.28 *P* value = 0.000001, 0.000002 &0.003336 respectively) upon comparing CD133+ cells separated from BM to those separated from PB of the LC group as shown in **S8 Table** and **Fig 7B.** On comparing CD133+ cells separated from BM of the LC group to those separated from BM of the control group *miR-122*, *miR-192*, *miR-224 miR-22 miR-101*, *miR-125a-5p*, *miR-29b* and *miR-199a-3p* were significantly down regulated (fold change = 0.34, 0.37, 0.54, 0.41, 0.31, 0.48, 0.6, 0.27 & 0.5 *P* value = 0.00015, 0.000046, 0.000249, 0.000287, 0.000001, 0.000397, 0.009098, 0.000004 & 0.000051 respectively) and only *miR-602* was significantly up regulated (fold change = 2.7 *P* value = 0.02) as shown in **S9 Table** and **Fig 7C**.

**Fig 8:**
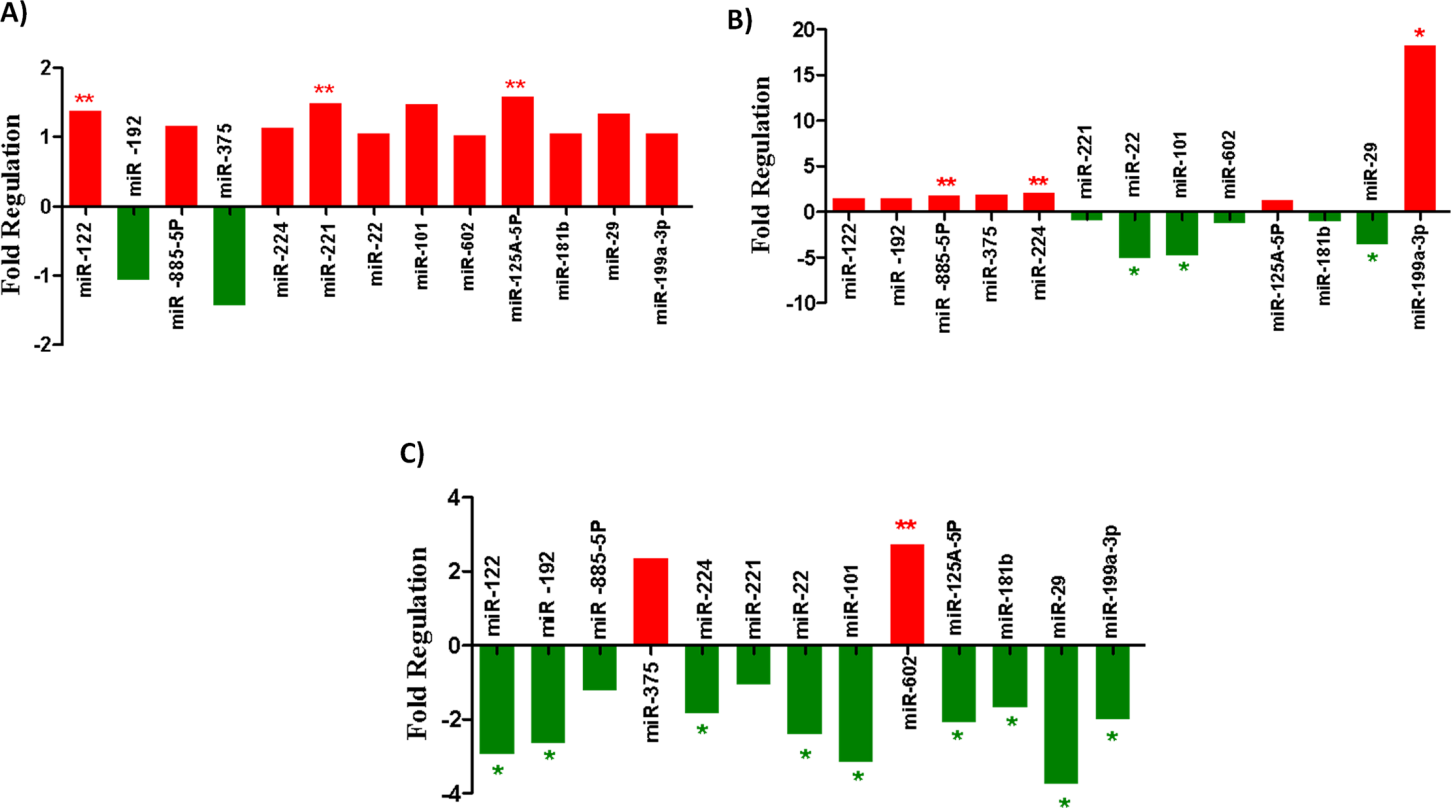
A) Histogram showing the differential expression of the 13 miRNAs in CD133+ cells of the control group (BM) versus the control group (PB). B) Histogram showing the differential expression of the 13 studied miRNAs in CD133+cells of the LC group (BM) versus the LC group (PB). C) Histogram showing the differential expression of the 13 miRNAs in CD133+ cells of the LC group (BM) versus the control group (BM). "*" miRNA is significant at 0.05 level while "**" miRNA is significant at 0.01 level.

The histograms representing the differential expression of miRNAs in CD133+ cells purified from bone marrow of the LC and control groups were shown in **Fig 8**.

### Confirmatory set results

**Fig 9:**
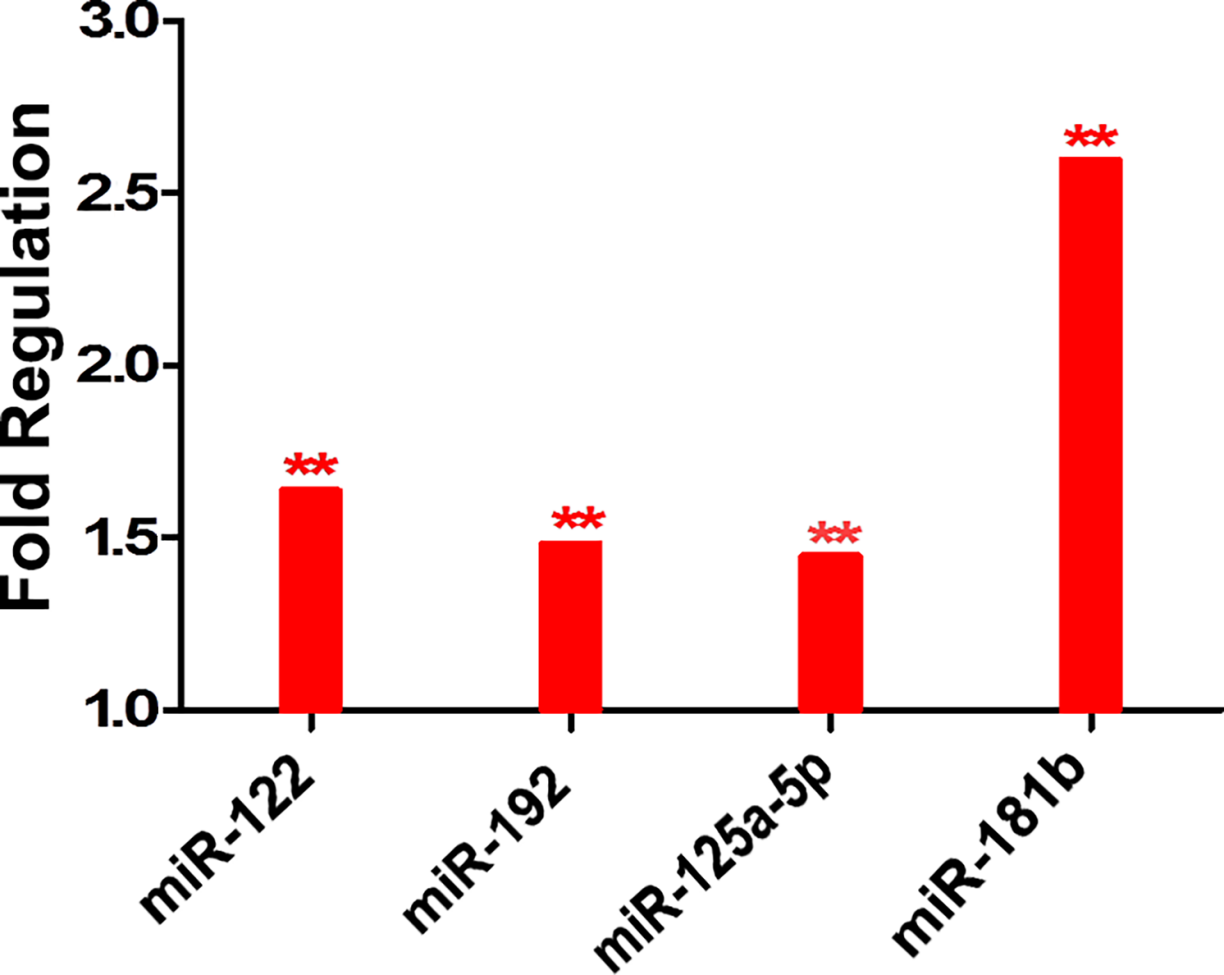
Histogram of the confirmatory set showing the differential expression of the four miRNAs in CD133+ cells of the HCC versus the control group (PB).

The differential expression of the studied miRNAs in CD133+ of the HCC group compared to the control group showed up regulation of miR-181b, miR-192, miR-125a-5p and miR-122 in concordance with the our previous data **Table 4, Fig 9.** Moreover, there is no significant difference in the miRNA expression between both methods used for CD133+ cell sorting.

**Table 4:** The differential expression of the studied miRNAs in the CD133+ cells of the HCC group versus the normal control group.

No	miR-name	Fold change	95% CI	*P* value
**1**	***miR-122***	**1.634**	**(1.33–1.93**	< 0.0001***
**2**	***miR -192***	**1.4798**	**(1.28–1.69**	< 0.0001***
**10**	***miR-125a-5P***	**1.4441**	**(1.21, 1.67)**	0.0004^***^
**11**	***miR -181b***	**2.593**	**(1.49–2.34**	< 0.0001***

## Discussion

Cancer stem cells (CSCs) represent a subset of cells that exist within the tumor. They are responsible for resistance, survival and recurrence after the treatment. CD133+ has drawn a significant attention as a critical liver CSC marker **[15].** There is evidence that miRNAs are significantly involved in self-renewal and differentiation of CSCs **[16]**. They are responsible for regulating the stemness characteristics through modulation of tumor-suppressive and oncogenic pathways **[17]**.

In this study, we aimed to differeniate between the naive circulating CD133 + cells obtained from patients at different stages of HCV-associated liver disease and the CD133 + cells of the healthy controls at the molecular level through comparing the expression patterns of 13 miRNAs.

Our results revealed that *miR-181b*, *miR-602*, *miR-101*, *miR-122*, *miR-192*, *miR-125a-5p miR-221* and *miR-22* were significantly up regulated up on comparing CD133+ cells of the HCC group (PB) to those of the control group (PB).

*MiR-181b* which belongs to *miR-181s* family; plays an important role in liver injury and cancer development **[18].** Significant up regulation of *miR-181b* was in concordance with a previous report by Ji et al. who found that multiple members of the *miR-181* family, including *miR-181b* are up-regulated in the liver CSC subset marked with EpCAM+ AFP+ **[19].** Similarly, our previous study addressed that *miR-181* was significantly up regulated in sera of the HCC group when compared to the LC group **[14].** Moreover, Meng et al identified the differential expression of *miR-181* family members in an Oct4 + CD133 + liver CSC subset. They also identified a critical role for miR-*181s* in Twist-driven metastasis in this Oct4 liver CSCs through targeting RAS-association domain family 1, isoform A (*RASSF1A*), tissue inhibitor of metalloproteinase 3 (*TIMP3*) and nemo-like kinase (*NLK*) **[18].** Furthermore, *miR-181s* was found to maintain stemness by directly targeting GATA-binding protein 6 (*GATA6*) caudal type homeobox 2 (*CDX2*) and nemo-like kinase (*NLK*) which is a potent inhibitor of β-catenin resulting in activation of the Wnt/β-catenin pathway in EpCAM + liver CSCs **[19].**

**Our data regarding the significant up regulation of *miR-602* was in agreement with yang et al. who identified the oncogenic function of *miR-602* in HCC through targeting the tumor suppressive function of *RASSF1A*** [20].

On the other hand, *miR-101* was reported to inhibit tumor aggressiveness through targeting EZH2 which is an epigenetic regulator of cell survival, proliferation, CSC phenotype and function, in various cancers including endometrial cancer **[21]** prostate cancer **[22]**, pancreatic cancer **[23]**. On contrary to these data, we found that *miR-101* was significantly up regulated. The possible explanation is that individual miRNA may act as oncomir or tumor suppressor according to its organ specificity.

Besides, *miR-122* and *miR-192* are the most two abundantly expressed miRNAs in the human liver **[24]**. *miR-122* represents 70% of the total miRNAs in the normal liver **[25,26].** In the primary HCC tissues as well as hepatoma cell lines, *miR-122* was found to be down-regulated conceiving its role as a tumor-suppressive miRNA **[27,28]**.It targets many oncogenic genes such as disintegrin and metalloprotease 17 (*ADAM17*) **[29]**, *Bcl-w* **[30]**, *Wnt1***[31]** and cyclin G1 (*CCGN1*) **[32]**. The significant up regulation of *miR-122* in the circulating CD133 cells purified from HCC patients associated with HCV infection may be attributed to that CD133 cells serve as a reservoir for HCV **[33]** which in turn stimulates the expression of *miR-122* in favor of promoting its genome replication and translation **[34].** Also, the exogenous expression of *miR-122* supports HCV replication and propagation in the non- permissive cell line **[35].** Similarly, our previous study reported that *miR-122* was significantly up regulated in the serum samples of HCC patients associated with HCV infection when compared to the healthy controls **[14]**. As for *miR-122*, *miR-192* also enhances HCV replication **[36].** Moreover, Yan-Chun *et al*., reported that *miR-192-5p* promotes the proliferation and metastasis of HCC expressing CD133 and CD90 cell surface markers through targeting semaphorin 3A (*SEMA3A*) **[37]**.

Additionally, *miR-125a* is frequently down-regulated in HCC and it inversely correlated with aggressiveness and poor prognosis of the tumor. Ectopic expression of *miR-125a* may down-regulate *MMP11* and *VEGF in vitro* and *in vivo* supporting its role as tumor suppressive miRNA; inhibiting the proliferation, invasion and metastasis of HCC **[38]**. Moreover, *miR-125a* inhibits the invasion of HCC cells through regulating the PI3K/AKT/mTOR pathway **[39].** However, the significant up-regulation of *miR-125a-5p* in this study came in agreement with an early report by Guo *et al*. who proved that *miR-125a* alone was capable of increasing the number of hematopoietic stem cells *in vivo* via targeting multiple proapoptotic genes e.g. *Bak1* **[40].** In addition, *ERBB2* and *ERBB3* were negatively regulated by *miR-125a* and *miR-125b*. In CD133+ cells, the down-regulation of *ERBB2* due to the effect of *miR-125* might lead to more polarized morphology. Therefore, *miR-125a* plays an anti apoptotic role and participates in remodeling of cytoskeleton and dedifferentiation **[41].**

*miR-221* is up regulated in 70%–80% of HCC samples with the maximum divergence affecting several cancer pathways **[42]**. Also, *miR-221* stimulates the onset of tumors, promotes tumor progression and shorts the time to death in the mouse model of liver cancer **[43]**. *miR-221* controls cell cycle progression, apoptosis, cell migration and stemness through targeting *CDKN1B* (*p27*) and *CDKN1C* (*p57*), *PUMA*, *FOXO3*, *PTEN*, *Bim*, *c-Kit*, *TIMP3*, *ER-α* and *DNMT3b* **[44–46].** In line with this, over expressing of *miR-221* enhances proliferation, migration, and invasion capability of the HCC cells **[42,47,48]**. Our previous study reported an up-regulation of *miR-221* in HCC patientsˋ sera compared to LC patients **[14].** We have previously reported a similar data regarding the up regulation *of miR-221* in HCC patientsˋ sera compared to LC patients.

On comparing CD133+ cells of the HCC group (PB) to those cells of the control group (PB); *miR-122*, *miR-192*, *miR-885-5p*, *miR-224*, *miR125a-5p*, *miR-181b* and *miR-199a-3p* were significantly up-regulated whereas *miR-22* was significantly down-regulated. Similarly, Gui et al. reported that *miR-122*, *miR-192*, *miR-885-5p* and *miR-224* were up regulated in the sera of HCC and LC patients compared to the normal controls **[49].** Moreover, Chen et al. showed that *miR-224* starts to be up regulated from the precancerous stage and its up regulation persists throughout the HCC development **[50]**. *miR-224* plays an important role in cell proliferation, migration, invasion, and prevention of apoptosis in HCC through its binding to target genes, such as *CDC42*, *CDH1*, *PAK2*, *BCL-2*, and *MAPK1* suggesting its role as an oncomir **[51,52].**

Down regulation of *miR-199a-3p* in HCC has been reported in various previous studies **[53–56]** supporting its function as a tumor suppressor miRNA. On the contrary, *miR-199a-3p* was reported to be up regulated and promote tumor progression in other cancer types **[57–60].** It also promotes the hematopoietic stem/ progenitor cells (HSPC) proliferation and plays a crucial role in hematopoiesis **[61].**

*MiR-22* also plays an important role in tumorigenesis **[62]**. The significant down regulation of *miR-22* was concordant with our previous data which reported its down regulated level in the sera of HCC patients compared to LC patients **[14]**. Also, Zang *et al*., reported that the decreased level of *miR-22* was associated with poor clinical outcome of liver cancer patients **[62]**. Several studies addressed that *miR-22* over-expression targeting *PTEN*, *p21*, and *p53* leads to reduction of cell growth, invasion, and metastasis in several cancer types **[62–65]**; supporting its tumor suppressive function. Furthermore, the proliferation of CD133+ is greatly affected by the miRNAs expression level itself. Such relation was reported by Zhang et al who showed that miR-150 over expression lead to a significant reduction of CD133+ cells, accompanied by significant inhibition of cell growth and tumorsphere formation. So, miR-150 may be involved in liver CSC self-renewal, potentially via modulation of the downstream target c-Myb **[66]**.

Upon comparing CD133+ cells of the HCC group (PB) to CD133+ cells of the CHC group (PB); *miR-181b*, *miR-122*, *miR-192* and *miR-125a-5p* were significantly up-regulated. Furthermore, *miR-122*, *miR-192*, *miR-125a-5p* and *miR-181b* were significantly common up-regulated miRNAs in CD133+ cells of the HCC group (malignant group) compared to the other non-malignant groups (CHC, LC and control groups).

To the best of our knowledge, this the first study showed the differential miRNAs expression pattern of CD133+ cells obtained from BM versus CD133+ cells obtained from PB of the same participant. We found that *miR-122*, *miR-221* and *miR-125a-5p* were significantly up-regulated on comparing CD133+ cells separated from BM to those separated from PB of the control group. However, *miR-885-5p*, *miR-224* and *miR-199a-3p* were significantly up-regulated whereas *miR-22*, *miR-101* and *miR-29b* were significantly down-regulated in the CD133+ cells separated from BM compared to those separated from PB of the LC group. On comparing the differential miRNAs expression pattern of CD133+ cells separated from BM of the LC group to those separated from BM of the control group vs. CD133+ cells separated from PB of the LC group to those separated from PB of the control group we found that *miR-122*, *miR-192*, *miR-199a-3p*, *miR-125a-5p* and *miR-224* were common significantly down regulated miRNAs. Further study will be needed to investigate the role of those miRNAs in the differentiation of CD133+ cells.

## Conclusion

These results have provided the evidence that the miRNAs differentially expressed between the CD133+ cells associated with HCC group associated with HCV infection and CD133+ cells of the other non-malignant studied groups. Those miRNAs are highly involved in stemness, proliferation, and remodeling of cytoskeleton and apoptosis blocking. These findings broaden our knowledge about the molecular characterization along with phenotypic characterization of CD133+ cells for stem cell-based therapies. However, there are some limitations in this study as phenotypic characterization with the double stained and clonogenic assays will be needed to prove the carcinogenic properties of the stem cells harboring CD133+ surface marker in HCC.

## Supporting information

S1 Table The differential expression of the 13 studied miRNAs in the CD133+ cells of the CHC group (PB) versus the control group (PB). (DOC)

S2 Table The differential expression of the 13 studied miRNAs in the CD133+ cells of the LC group (PB) versus the control group (PB). (DOC)

S3 Table The differential expression of the 13 studied miRNAs in the CD133+ cells of the HCC group (PB) versus the control group (PB). (DOC)

S4 Table The differential expression of the 13 studied miRNAs in purified CD133+ cells in HCC group (PB) versus the CHC group (PB). (DOC)

S5 Table The differential expression of the 13 studied miRNAs in the CD133+ cells of the HCC group (PB) versus the LC group (PB). (DOC)

S6 Table The differential expression of the 13 studied miRNAs in the CD133+ cells of the HCC group (PB) versus all studied non-malignant groups (PB). (DOC)

S7 Table The differential expression of the 13 studied miRNAs in the CD133+ cells of the control group (PB) versus the control group (BM). (DOC)

S8 Table The differential expression of the 13 studied miRNAs in the CD133+ cells of the LC group (PB) versus the LC group (BM). (DOC)

S9 Table The differential expression of the 13 studied miRNAs in the CD133+ cells of the group (BM) versus the control group (BM). (DOC)
