# Supplementary material for: MicroRNA Signatures for circulating CD133-positive cells in hepatocellular carcinoma with HCV infection
Source: PLoS One. 2018 Mar 13;13(3):e0193709. doi: 10.1371/journal.pone.0193709 (PMC5849309; doi:10.1371/journal.pone.0193709)
Supplement: S1 Table — (DOC) [file pone.0193709.s001.doc]

**S1 Table:** The differential expression of the 13 studied miRNAs in the CD133+ cells of the CHC group (PB) versus the control group (PB).

| **No** | **miR-name** | **Fold change** | **Fold regulation** | **95% CI** | ***P* value** |
| --- | --- | --- | --- | --- | --- |
| **1** | ***miR-122*** | **0.573** | **-1.7451** | **( 0.44, 0.70 )** | **0.001404^a^** |
| **2** | ***miR -192*** | 1.093 | 1.093 | ( 1.01, 1.18 ) | 0.084445 |
| **3** | ***miR -885-5P*** | 0.9937 | -1.0064 | ( 0.85, 1.14 ) | 0.964445 |
| **4** | ***miR -375*** | 3.4204 | 3.4204 | ( 0.00001, 7.46 ) | 0.124131 |
| **5** | ***miR -224*** | 1.0855 | 1.0855 | ( 0.77, 1.40 ) | 0.721497 |
| **6** | ***miR -221*** | 1.2592 | 1.2592 | ( 1.04, 1.48 ) | 0.055444 |
| **7** | ***miR -22*** | 1.21 | 1.21 | ( 0.92, 1.50 ) | 0.15095 |
| **8** | ***miR -101*** | **1.6974** | **1.6974** | **( 1.05, 2.35 )** | **0.011783^b^** |
| **9** | ***miR -602*** | 0.4171 | -2.3977 | ( 0.00001, 1.14 ) | 0.94123 |
| **10** | ***miR -125a-5P*** | 0.9971 | -1.0029 | ( 0.67, 1.32 ) | 0.851383 |
| **11** | ***miR -181b*** | 1.0395 | 1.0395 | ( 0.82, 1.25 ) | 0.673143 |
| **12** | ***miR -29b*** | 1.2226 | 1.2226 | ( 0.58, 1.87 ) | 0.757493 |
| **13** | ***miR -199a-3p*** | 0.8827 | -1.1329 | ( 0.66, 1.11 ) | 0.550772 |

**^a^ miRNA is significant at 0.01 level**

**^b^ miRNA is significant at 0.05 level**
