# Supplementary material for: MicroRNA Signatures for circulating CD133-positive cells in hepatocellular carcinoma with HCV infection
Source: PLoS One. 2018 Mar 13;13(3):e0193709. doi: 10.1371/journal.pone.0193709 (PMC5849309; doi:10.1371/journal.pone.0193709)
Supplement: S2 Table — (DOC) [file pone.0193709.s002.doc]

**S2 Table: The differential expression of the 13 studied miRNAs in the CD133+ cells of the LC group (PB) versus the control group (PB).**

| **No** | **miR-name** | **Fold change** | **Fold regulation** | **95% CI** | ***P* value** |
| --- | --- | --- | --- | --- | --- |
| **1** | ***miR-122*** | **0.3572** | **-2.7992** | **( 0.12, 0.60 )** | **0.015476^b^** |
| **2** | ***miR -192*** | **0.2538** | **-3.9404** | **( 0.03, 0.47 )** | **0.00569^a^** |
| **3** | ***miR -885-5P*** | **0.5494** | **-1.8203** | **( 0.50, 0.60 )** | **0.000001^a^** |
| **4** | ***miR -375*** | 1.4854 | 1.4854 | ( 0.00001, 3.61 ) | 0.422176 |
| **5** | ***miR -224*** | **0.3349** | **-2.9862** | **( 0.12, 0.55 )** | **0.0111^b^** |
| **6** | ***miR -221*** | 1.5044 | 1.5044 | ( 0.79, 2.22 ) | 0.170911 |
| **7** | ***miR -22*** | **2.4852** | **2.4852** | **( 1.93, 3.04 )** | **0.000002^a^** |
| **8** | ***miR -101*** | **2.7352** | **2.7352** | **( 1.65, 3.82 )** | **0.000233^a^** |
| **9** | ***miR -602*** | 4.5002 | 4.5002 | ( 0.00001, 9.86 ) | 0.095591 |
| **10** | ***miR -125a-5P*** | **0.6647** | **-1.5044** | **( 0.46, 0.87 )** | **0.036711 ^b^** |
| **11** | ***miR -181b*** | 0.701 | -1.4265 | ( 0.23, 1.17 ) | 0.787193 |
| **12** | ***miR -29b*** | 1.6339 | 1.6339 | ( 0.57, 2.69 ) | 0.225416 |
| **13** | ***miR -199a-3p*** | **0.03** | **-33.2819** | **( 0.00001, 0.09 )** | **0.000009 ^b^** |

**^a^ miRNA is significant at 0.01 level**

**^b^ miRNA is significant at 0.05 level**
