# Supplementary material for: MicroRNA Signatures for circulating CD133-positive cells in hepatocellular carcinoma with HCV infection
Source: PLoS One. 2018 Mar 13;13(3):e0193709. doi: 10.1371/journal.pone.0193709 (PMC5849309; doi:10.1371/journal.pone.0193709)
Supplement: S3 Table — (DOC) [file pone.0193709.s003.doc]

**S3 Table: The differential expression of the 13 studied miRNAs in the CD133+ cells of the HCC group (PB) versus the control group (PB).**

| **No** | **miR-name** | **Fold change** | **Fold regulation** | **95% CI** | ***P* value** |
| --- | --- | --- | --- | --- | --- |
| **1** | ***miR-122*** | **1.8025** | **1.8025** | **( 1.13, 2.48 )** | **0.039774^b^** |
| **2** | ***miR -192*** | **1.7151** | **1.7151** | **( 1.45, 1.98 )** | **0.001903^a^** |
| **3** | ***miR-885-5P*** | 1.2901 | 1.2901 | ( 0.98, 1.60 ) | 0.12236 |
| **4** | ***miR -375*** | 5.5117 | 5.5117 | ( 0.00001, 12.35 ) | 0.074525 |
| **5** | ***miR -224*** | 1.0222 | 1.0222 | ( 0.72, 1.32 ) | 0.94376 |
| **6** | ***miR -221*** | **1.5994** | **1.5994** | **( 1.42, 1.77 )** | **0.000007^a^** |
| **7** | ***miR -22*** | 1.434 | 1.434 | ( 1.04, 1.83 ) | 0.063682 |
| **8** | ***miR -101*** | **2** | **2** | **( 1.24, 2.76 )** | **0.0013^a^** |
| **9** | ***miR -602*** | **5.4264** | **5.4264** | **( 0.00001, 10.89 )** | **0.037211^b^** |
| **10** | ***miR-125a-5P*** | **1.6808** | **1.6808** | **( 1.37, 1.99 )** | **0.000241^a^** |
| **11** | ***miR -181b*** | **2.9914** | **2.9914** | **( 2.48, 3.51 )** | **0.000044^a^** |
| **12** | ***miR -29b*** | 1.7942 | 1.7942 | ( 0.85, 2.74 ) | 0.053732 |
| **13** | ***miR -199a-3p*** | 1.1892 | 1.1892 | ( 1.02, 1.36 ) | 0.068102 |

**^a^ miRNA is significant at 0.01 level**

**^b^ miRNA is significant at 0.05 level**
