# Supplementary material for: MicroRNA Signatures for circulating CD133-positive cells in hepatocellular carcinoma with HCV infection
Source: PLoS One. 2018 Mar 13;13(3):e0193709. doi: 10.1371/journal.pone.0193709 (PMC5849309; doi:10.1371/journal.pone.0193709)
Supplement: S4 Table — (DOC) [file pone.0193709.s004.doc]

**S4 Table:** The differential expression of the 13 studied miRNAs in purified CD133+ cells in HCC group (PB) versus the CHC group (PB).

| **No** | **miR-name** | **Fold Change** | **Fold Regulation** | **95% CI** | ***P* value** |
| --- | --- | --- | --- | --- | --- |
| **1** | ***miR-122*** | **3.1456** | **3.1456** | **( 2.00, 4.29 )** | **0.001051^a^** |
| **2** | ***miR -192*** | **1.5692** | **1.5692** | **( 1.32, 1.82 )** | **0.000731^a^** |
| **3** | ***miR -885-5P*** | 1.2983 | 1.2983 | ( 0.95, 1.65 ) | 0.077361 |
| **4** | ***miR -375*** | 1.6114 | 1.6114 | ( 0.00001, 3.41 ) | 0.286042 |
| **5** | ***miR -224*** | -1.0619 | -1.0619 | ( 0.87, 1.02 ) | 0.187286 |
| **7** | ***miR -221*** | 1.2702 | 1.2702 | ( 0.06, 1.3 ) | 0.071281 |
| **8** | ***miR -22*** | 1.1851 | 1.1851 | ( 0.96, 1.41 ) | 0.107085 |
| **9** | ***miR -101*** | 1.1783 | 1.1783 | ( 0.95, 1.23 ) | 0.090025 |
| **10** | ***miR -602*** | **13.0111** | **13.0111** | **( 0.00001, 33.97 )** | **0.01257^b^** |
| **11** | ***miR -125a-5P*** | **1.6857** | **1.6857** | **( 1.20, 2.17 )** | **0.002189 ^a^** |
| **12** | ***miR -181b*** | **2.8779** | **2.8779** | **( 2.25, 3.50 )** | **0.000004 ^a^** |
| **13** | ***miR -29b*** | 1.4675 | 1.4675 | ( 0.88, 1.57 ) | 0.162006 |
| **14** | ***miR -199a-3p*** | 1.3472 | 1.3472 | ( 0.99, 1.70 ) | 0.05144 |

**^a^ miRNA is significant at 0.01 level**

**^b^ miRNA is significant at 0.05 level**
