# Supplementary material for: MicroRNA Signatures for circulating CD133-positive cells in hepatocellular carcinoma with HCV infection
Source: PLoS One. 2018 Mar 13;13(3):e0193709. doi: 10.1371/journal.pone.0193709 (PMC5849309; doi:10.1371/journal.pone.0193709)
Supplement: S5 Table — (DOC) [file pone.0193709.s005.doc]

**S5 Table:** The differential expression of the 13 studied miRNAs in the CD133+ cells of the HCC group (PB) versus the LC group (PB).

| **No** | **miR-name** | **Fold change** | **Fold regulation** | **95%CI** | ***P* value** |
| --- | --- | --- | --- | --- | --- |
| **1** | ***miR-122*** | **5.0455** | **5.0455** | **( 1.33, 8.76 )** | **0.001025 ^a^** |
| **2** | ***miR -192*** | **6.7583** | **6.7583** | **( 0.85, 12.67 )** | **0.000024 ^a^** |
| **3** | ***miR -885-5P*** | **2.3484** | **2.3484** | **( 1.79, 2.91 )** | **0.000472 ^a^** |
| **4** | ***miR -375*** | 3.7106 | 3.7106 | ( 0.00001, 8.81 ) | 0.102204 |
| **5** | ***miR -224*** | **3.0525** | **3.0525** | **( 1.27, 4.83 )** | **0.000278 ^a^** |
| **6** | ***miR -221*** | 1.0631 | 1.0631 | ( 0.57, 1.56 ) | 0.756324 |
| **7** | ***miR -22*** | **0.577** | **-1.7331** | **( 0.48, 0.68 )** | **0.000022 ^a^** |
| **8** | ***miR -101*** | 0.7312 | -1.3676 | ( 0.65, 1.21 ) | 0.058937 |
| **9** | ***miR -602*** | 1.2058 | 1.2058 | ( 0.00001, 2.44 ) | 0.953288 |
| **10** | ***miR-125a-5P*** | **2.5286** | **2.5286** | **( 1.85, 3.21 )** | **0.000004 ^a^** |
| **11** | ***miR -181b*** | **4.2673** | **4.2673** | **( 1.37, 7.16 )** | **0.000075 ^a^** |
| **12** | ***miR -29b*** | 1.0981 | 1.0981 | ( 0.67, 1.52 ) | 0.987949 |
| **13** | ***miR-199a-3p*** | **39.5791** | **39.5791** | **( 0.00001, 118.99 )** | **0.0000001 ^a^** |

**^a^ miRNA is significant at 0.01 level**

**^b^ miRNA is significant at 0.05 level**
