# Supplementary material for: MicroRNA Signatures for circulating CD133-positive cells in hepatocellular carcinoma with HCV infection
Source: PLoS One. 2018 Mar 13;13(3):e0193709. doi: 10.1371/journal.pone.0193709 (PMC5849309; doi:10.1371/journal.pone.0193709)
Supplement: S6 Table — (DOC) [file pone.0193709.s006.doc]

**S6 Table:** The differential expression of the 13 studied miRNAs in the CD133+ cells of the HCC group (PB) versus all studied non-malignant groups (PB).

| **No** | **miR-name** | **Fold change** | **Fold regulation** | **95%CI** | ***P* value** |
| --- | --- | --- | --- | --- | --- |
| **1** | ***miR-122*** | **2.9794** | **2.9794** | **( 1.63, 4.33 )** | **0.000013** |
| **2** | ***miR -192*** | **2.6188** | **2.6188** | **( 1.46, 3.78 )** | **0.000024** |
| **3** | ***miR -885-5P*** | 1.5637 | 1.5637 | ( 1.14, 1.99 ) | 0.177934 |
| **4** | ***miR -375*** | 2.7143 | 2.7143 | ( 0.02, 5.40 ) | 0.311972 |
| **5** | ***miR -224*** | 1.3734 | 1.3734 | ( 0.91, 1.84 ) | 0.457634 |
| **6** | ***miR -221*** | 1.2662 | 1.2662 | ( 1.05, 1.49 ) | 0.333491 |
| **7** | ***miR -22*** | 0.9578 | -1.0441 | ( 0.72, 1.20 ) | 0.586973 |
| **8** | ***miR -101*** | 1.1181 | 1.1181 | ( 0.90, 1.34 ) | 0.814632 |
| **9** | ***miR -602*** | 4.0919 | 4.0919 | ( 0.29, 7.89 ) | 0.091346 |
| **10** | ***miR-125a-5P*** | **1.8754** | **1.8754** | **( 1.54, 2.21 )** | **0.000013** |
| **11** | ***miR -181b*** | **3.2565** | **3.2565** | **( 2.38, 4.13 )** | **0.000001** |
| **12** | ***miR -29b*** | 1.3079 | 1.3079 | ( 1.04, 1.57 ) | 0.281099 |
| **13** | ***miR-199a-3p*** | 3.9252 | 3.9252 | ( 0.00001, 7.88 ) | 0.158915 |

**^a^ miRNA is significant at 0.01 level**

**^b^ miRNA is significant at 0.05 level**
