# Supplementary material for: MicroRNA Signatures for circulating CD133-positive cells in hepatocellular carcinoma with HCV infection
Source: PLoS One. 2018 Mar 13;13(3):e0193709. doi: 10.1371/journal.pone.0193709 (PMC5849309; doi:10.1371/journal.pone.0193709)
Supplement: S7 Table — (DOC) [file pone.0193709.s007.doc]

**S7 Table:** The differential expression of the 13 studied miRNAs in the CD133+ cells of the control group (PB) versus the control group (BM).

| **No** | **miR-name** | **Fold change** | **Fold regulation** | **95%CI** | ***P* value** |
| --- | --- | --- | --- | --- | --- |
| **1** | ***miR-122*** | **1.3708** | **1.3708** | **( 1.00, 1.74 )** | **0.044214^b^** |
| **2** | ***miR -192*** | 0.9374 | -1.0668 | ( 0.83, 1.05 ) | 0.33556 |
| **3** | ***miR -885-5P*** | 1.154 | 1.154 | ( 0.89, 1.41 ) | 0.190072 |
| **4** | ***miR -375*** | 0.699 | -1.4306 | ( 0.00001, 1.65 ) | 0.549156 |
| **5** | ***miR -224*** | 1.1238 | 1.1238 | ( 0.80, 1.45 ) | 0.51295 |
| **6** | ***miR -221*** | **1.4914** | **1.4914** | **( 1.34, 1.64 )** | **0.000004^b^** |
| **7** | ***miR -22*** | 1.0534 | 1.0534 | ( 0.75, 1.35 ) | 0.705273 |
| **8** | ***miR -101*** | 1.4709 | 1.4709 | ( 0.95, 1.99 ) | 0.063632 |
| **9** | ***miR -602*** | 1.0163 | 1.0163 | ( 0.00001, 2.06 ) | 0.750873 |
| **10** | ***miR-125a-5P*** | **1.5801** | **1.5801** | **( 1.26, 1.90 )** | **0.001312 ^b^** |
| **11** | ***miR -181b*** | 1.0485 | 1.0485 | ( 0.92, 1.18 ) | 0.501508 |
| **12** | ***miR -29b*** | 1.3287 | 1.3287 | ( 0.70, 1.96 ) | 0.454611 |
| **13** | ***miR-199a-3p*** | 1.0473 | 1.0473 | ( 0.87, 1.22 ) | 0.5323 |

**^a^ miRNA is significant at 0.01 level**

**^b^ miRNA is significant at 0.05 level**
