# Supplementary material for: MicroRNA Signatures for circulating CD133-positive cells in hepatocellular carcinoma with HCV infection
Source: PLoS One. 2018 Mar 13;13(3):e0193709. doi: 10.1371/journal.pone.0193709 (PMC5849309; doi:10.1371/journal.pone.0193709)
Supplement: S8 Table — (DOC) [file pone.0193709.s008.doc]

**S8 Table:** The differential expression of the 13 studied miRNAs in the CD133+ cells of the LC group (PB) versus the LC group (BM).

| **No** | **miR-name** | **Fold change** | **Fold regulation** | **95%CI** | ***P* value** |
| --- | --- | --- | --- | --- | --- |
| **1** | ***miR-122*** | 1.4077 | 1.4077 | ( 0.47, 2.34 ) | 0.743741 |
| **2** | ***miR -192*** | 1.4109 | 1.4109 | ( 0.10, 2.72 ) | 0.995331 |
| **3** | ***miR -885-5P*** | **1.7451** | **1.7451** | **( 0.91, 2.58 )** | **0.048477^b^** |
| **4** | ***miR -375*** | 1.8129 | 1.8129 | ( 0.00001, 4.05 ) | 0.784683 |
| **5** | ***miR -224*** | **2.0515** | **2.0515** | **( 0.85, 3.26 )** | **0.031087^b^** |
| **6** | ***miR -221*** | 0.9885 | -1.0116 | ( 0.38, 1.59 ) | 0.876575 |
| **7** | ***miR -22*** | **0.1957** | **-5.11** | **( 0.17, 0.23 )** | **0.000001^a^** |
| **8** | ***miR -101*** | **0.209** | **-4.7844** | **( 0.14, 0.28 )** | **0.000002 ^a^** |
| **9** | ***miR -602*** | 0.7658 | -1.3059 | ( 0.07, 1.46 ) | 0.229321 |
| **10** | ***miR-125a-5P*** | 1.234 | 1.234 | ( 0.77, 1.70 ) | 0.276395 |
| **11** | ***miR -181b*** | 0.9428 | -1.0607 | ( 0.24, 1.65 ) | 0.502329 |
| **12** | ***miR -29b*** | **0.28** | **-3.5718** | **( 0.14, 0.42 )** | **0.003336 ^a^** |
| **13** | ***miR-199a-3p*** | **18.1891** | **18.1891** | **( 0.00001, 54.64 )** | **0.000091 ^a^** |

**^a^ miRNA is significant at 0.01 level**

**^b^ miRNA is significant at 0.05 level**
