# Supplementary material for: MicroRNA Signatures for circulating CD133-positive cells in hepatocellular carcinoma with HCV infection
Source: PLoS One. 2018 Mar 13;13(3):e0193709. doi: 10.1371/journal.pone.0193709 (PMC5849309; doi:10.1371/journal.pone.0193709)
Supplement: S9 Table — (DOC) [file pone.0193709.s009.doc]

**S9 Table:** The differential expression of the 13 studied miRNAs in the CD133+ cells of the LC group (BM) versus the control group (BM).

| **No** | **miR-name** | **Fold change** | **Fold regulation** | **95%CI** | ***P* value** |
| --- | --- | --- | --- | --- | --- |
| **1** | ***miR-122*** | **0.3392** | **-2.9485** | **( 0.25, 0.42 )** | **0.000153^a^** |
| **2** | ***miR -192*** | **0.3772** | **-2.6512** | **( 0.24, 0.51 )** | **0.000046 ^a^** |
| **3** | ***miR -885-5P*** | 0.8076 | -1.2383 | ( 0.38, 1.23 ) | 0.677485 |
| **4** | ***miR -375*** | 2.3376 | 2.3376 | ( 0.00001, 5.01 ) | 0.26579 |
| **5** | ***miR -224*** | **0.539** | **-1.8553** | **( 0.44, 0.64 )** | **0.000249^a^** |
| **6** | ***miR -221*** | 0.9374 | -1.0668 | ( 0.56, 1.31 ) | 0.861094 |
| **7** | ***miR -22*** | **0.4137** | **-2.4172** | **( 0.31, 0.52 )** | **0.000287 ^a^** |
| **8** | ***miR -101*** | **0.3153** | **-3.1711** | **( 0.22, 0.41 )** | **0.000001 ^a^** |
| **9** | ***miR -602*** | **2.7289** | **2.7289** | **( 0.28, 5.18 )** | **0.020538 ^b^** |
| **10** | ***miR-125a-5P*** | **0.478** | **-2.0922** | **( 0.33, 0.62 )** | **0.000397 ^a^** |
| **11** | ***miR -181b*** | **0.5925** | **-1.6876** | **( 0.39, 0.80 )** | **0.009098 ^a^** |
| **12** | ***miR -29b*** | **0.2664** | **-3.7538** | **( 0.17, 0.36 )** | **0.000004 ^a^** |
| **13** | ***miR-199a-3p*** | **0.4977** | **-2.0093** | **( 0.42, 0.57 )** | **0.000051 ^a^** |

**^a^ miRNA is significant at 0.01 level**

**^b^ miRNA is significant at 0.05 level**
